# Patterns of c-reactive protein predicts response to therapy in healthcare-associated pneumonia in critically ill patients with cancer

**DOI:** 10.1186/2197-425X-3-S1-A254

**Published:** 2015-10-01

**Authors:** L Sarmet Cunha Farah Rabello, T Lisboo, L Azevedo, JR Lapa e Silva, M Soares, P Póvoa, J Salluh

**Affiliations:** Instituto Nacional de Cancer, Rio de Janeiro, Brazil; D'Or Institute for Research and Education, Rio de Janeiro, Brazil; Intensive Care Unit, Santa Casa de Misericórdia de Porto Alegre, Porto Alegre, Brazil; Intensive Care Unit, Hospital Sirio-Libanes, São Paulo, Brazil; Postgraduate Program of Internal Medicine, Universidade Federal do Rio de Janeiro, Rio de Janeiro, Brazil; Unidade Polivalente de Terapia Intensiva, Hospital de São Francisco Xavier, Centro Hospitalar de Lisboa Ocidental, CEDOC, Faculdade Médica NOVA, Nova Universidade de Lisboa, Lisboa, Portugal

## Intr

Pneumonia is the most frequent type of infection in cancer patients and is associated with exceedingly high mortality rates. Clinical judgment is insufficient to an early identification of outcomes in critically ill.

## Objectives

The aim of the present study was to evaluate the patterns of CRP response to antibiotic therapy during the first week in the ICU in cancer patients admitted with healthcare-associated pneumonia (HCAP).

## Methods

This was a secondary analysis of a prospective cohort of cancer patients admitted to three ICUs with the diagnosis of HCAP. CRP was sampled every other day from D0 do D6 of antibiotic prescription. Patients were classified according to an individual pattern of CRP-ratio response with the following criteria: fast response - when CRP at day 4 of therapy was < 0.4 of day 0 CRP; slow response - characterized by a continuous but slow decreased of CRP; non-response - when CRP remained always above 0.8 of day 0 CRP; biphasic response - characterized by an initial CRP decrease to levels < 0.8 of the day 0 CRP followed by a secondary rise > 0.8.

## Results

A total of 129 patients were included in the study (median age: 65 years; solid tumors 69%; neutropenia 13%). Good performance status was observed in 62%. The median Charlson comorbidity index was 3 points. Septic shock upon ICU admission was present in 74% of all patients, invasive mechanical ventilation was used in 73% and 27% used dialysis. ICU and hospital mortality rates were 47% and 64%, respectively. Microbiological confirmation was present in 51% of all patients, with a slight predominance of Gram-negative bacteria. A relatively low incidence of multiresistant pathogens (17%) was observed. 31 patients were classified as fast response pattern, 44 as slow response, 39 as nonresponse and 15 as biphasic response. The time-dependent analysis of relative variations of CRP of the four different patterns evolution was statistically different (p < 0.001). The ICU mortality rate was significantly different according to the patterns of response, fast response 12.9%, slow response 43.2%, biphasic response 66.7% and nonresponse 71.8% (p < 0.001).

## Conclusions

Serial evaluation of CRP ratio was useful in the early identification of cancer patients with HCAP with a poor outcome. Besides, the recognition of the patterns of CRP ratio in critically ill patients with cancer could significantly influence the clinical decision-making process at the bedside.

